# Qualitative evaluation of the Saleema campaign to eliminate female genital mutilation and cutting in Sudan

**DOI:** 10.1186/s12978-018-0470-2

**Published:** 2018-02-17

**Authors:** Andrea C. Johnson, W. Douglas Evans, Nicole Barrett, Howida Badri, Tamador Abdalla, Cody Donahue

**Affiliations:** 1 Milken Institute School of Public Health, The George Washington University, 950 New Hampshire Avenue, NW, Washington, DC 20052 USA; 2 UNICEF, Kassala, Sudan

**Keywords:** Female genital mutilation and cutting, Social marketing, Branding, Social norms, Focus groups, Qualitative research

## Abstract

**Background:**

Female genital mutilation and cutting (FGM/C, herein FGM) is a widespread and harmful practice. The Government developed a national campaign in Sudan, called Saleema, to change social norms discouraging FGM. Saleema translates to being “whole”, healthy in body and mind, unharmed, intact, pristine, and untouched, in a God-given condition. An interim evaluation was conducted using focus groups among Sudanese adults. The primary aim was to explore perceptions of the Saleema poster exemplars and to assess if the desired themes were being communicated. Secondary aims were to understand more about participants’ information sources, values, and suggestions for the campaign broadly.

**Methods:**

The Saleema campaign evaluation included four focus groups from each of the 18 states in Sudan (72 total). Participants were presented with three poster stimuli from the Saleema campaign and asked about the content and their reactions. Themes were coded inductively by concepts that arose through content in the transcripts. Codes were also reviewed in conjunction with themes from the broader Saleema evaluation framework.

**Results:**

Participants reported the most common source of information or admiration was from local leaders who are responsive to a community, media-based outlets, and discussions among community members. Participants held high value for education, community solidarity, and/or religious devotion. Participants had positive opinions of Saleema and responded positively to the branding elements in the posters and the campaign as a whole. The most common suggestion was continued awareness. Advocacy, training, and posters were suggested to highlight the harms of FGM through leaders or in community settings. Individuals suggested that these activities target older women and individuals in rural villages. There was also a burgeoning theme of targeting youth for support of the campaign.

**Discussion:**

The results of this focus group analysis demonstrate support for future Saleema campaign efforts promoting awareness and community engagement. The campaign could capitalize on partnerships with young people and those who are respected in the community (e.g., religious leaders) or continue promoting common values aligning with the support of education and community solidarity. Continuing campaign efforts have promise to decrease the harms of FGM in Sudan.

**Electronic supplementary material:**

The online version of this article (10.1186/s12978-018-0470-2) contains supplementary material, which is available to authorized users.

## Plain English summary

Female genital mutilation and cutting (FGM/C, herein FGM) is a widespread and harmful practice. The Government developed a national campaign in Sudan, called Saleema, to change social norms discouraging FGM. Saleema translates to being “whole”, healthy in body and mind, unharmed, intact, pristine, and untouched, in a God-given condition. The Saleema campaign evaluation included four focus groups within 18 states in Sudan (72 total). Participants were presented with three poster stimuli from the Saleema campaign and asked about the content and their reactions. The primary aim was to explore perceptions of the Saleema poster exemplars and to assess if the desired themes were being communicated. Secondary aims were to understand more about participants’ information sources, values, and suggestions for the campaign broadly. Participants had positive opinions of Saleema and responded positively to the branding elements in the posters and the campaign as a whole. The most common suggestion was continued awareness. Advocacy, training, and posters were suggested to highlight the harms of FGM through leaders or in community settings. Individuals suggested that these activities target older women and individuals in rural villages. There was also a burgeoning theme of targeting youth for support of the campaign. The results of this focus group analysis demonstrate support for future Saleema campaign efforts promoting awareness and community engagement. The campaign could capitalize on partnerships with young people and those who are respected in the community (e.g., religious leaders) or continue promoting common values aligning with the support of education and community solidarity. Continuing campaign efforts have promise to decrease the harms of FGM in Sudan.

## Background

The World Health Organization (WHO) and other global health and development organizations, including the Department for International Development (DFID) and US Agency for International Development (USAID), note female genital mutilation and cutting (FGM/C, herein FGM) is a widespread and harmful practice [1]. There are 4 main types of FGM, ranging in severity from Type I to IV [2]. FGM prevalence is highest in 27 countries in Africa and the Middle East [2, 3]. In Sudan, FGM is highly prevalent among all age groups of girls and women, with a national prevalence rate of 87% [4].

There is a steady, though modest, decline among the younger age cohorts (age 25 and below) and 52% of women believe the practice should stop [4]. The social practice of FGM has deep cultural and historical roots and has connotations with religious practice. When a social norm such as FGM is in place, families and individuals engage in the practice because they believe that it is expected of them and is prevalent. Without these perceptions, the social norm would be weakened and practice would become less widespread and may eventually cease to exist. Changing social norms is thus a key step in behavior change [5].

Health communication and branding is a common intervention modality to change social norms. The study team developed a national campaign in Sudan, called Saleema, to change social norms discouraging FGM. Saleema translates to being “whole” (e.g., physically, emotionally). The campaign uses health branding theory (HBT) [6]. In part, HBT holds that creating positive mental associations with the alternative to unhealthy or anti-social behavior promotes behavior change [6–8]. There is evidence that creating positive brand identifications, or brand equity, mediates the effects of interventions on behavior change [9]. After formative testing, campaign messaging and activities targeted the entire population including all age groups using an expansive branding strategy. The campaign primarily focused on messages of community cohesion, strength, and change to reject FGM norms. The campaign held local events in each state and used a variety of channels, such as the radio, television, and billboards to show posters communicating the various messages.

Within the longitudinal, mixed methods campaign evaluation, the authors conducted a qualitative analysis using focus groups among Sudanese adults. The primary aim was to explore perceptions of the Saleema poster exemplars and to assess if the desired themes were being communicated as well as if there was any identification with the Saleema brand. Secondary aims were to understand more about participants’ information sources, values, and suggestions for the campaign broadly.

## Methods

### Setting and materials

The Saleema campaign evaluation plan is a longitudinal mixed methods design. For the first round of the qualitative component, the evaluation included four nationally representative focus groups from each of the 18 states in Sudan using a pre-defined sampling framework. Following a cluster randomized sample plan, the study team randomly sampled two administrative units within each state to collect a sample [10]. The focus groups for this study were sampled using a Primary Sampling Unit (PSU) and randomly sampled from households in the same PSU. As a result, each state included focus groups in two geographically separate localities. Within each locality, there was a focus group composed of Sudanese adult males and females. Focus group moderators completed training and followed a focus group guide designed by the study team and UNICEF partners.

Participants were presented with three graphical poster stimuli from the Saleema campaign used in print advertisements and asked about the content and their reactions. The three posters represented common themes designed for the Saleema campaign and included bright red and green colors to represent those who supported the campaign. The Saleema campaign was branded with positive, gain-frame messaging, with themes focusing on changing norms and preventing FGM by way of choice and community cohesion. The first poster, “I am not afraid of change” represented changing norms within a community, showcasing a lack of fear in progress away from FGM. In particular, it seeks inclusivity of family members and community irrespective of age and sex. The second poster, “Because I am strong in my decision”, represents a young woman taking a stand against FGM and stating her desire to remain uncircumcised. Lastly, the third poster “Saleema” includes a circumcised woman holding her infant daughter. The mother is happy because she is choosing to not circumcise her daughter, thereby changing the trend of FGM in her family for the next generation.

The focus group guide also inquired about information sources and values. Importantly, questions also asked about support of and suggestions for the campaign. Focus group questions were designed using a conceptual framework drawn from common health behavior theories. Specifically, this included Health Branding Theory (e.g., brand equity) and Social Marketing principles (e.g., product, price, place, promotion) [6, 11]. This structure sought to assist in organizing suggestions around message content, channels for delivery, target populations, and methods of promotion for greater receptivity.

### Analysis and validation

All focus groups were recorded, transcribed, and translated by a team from Ahfad University from Arabic to English. This step was completed by a UNICEF staff member in Sudan. Transcript coding was conducted in an iterative fashion by a primary coder (ACJ) who was independent of the study design and data collection. Analysis was broadly organized by question in the focus group guide (e.g., information sources and values, campaign engagement, poster reactions, campaign suggestions). The focus group transcripts were coded first using axial coding where codes were provided “in vivo” labels, or words participants used [12]. As new transcripts were reviewed, the codes were iteratively grouped together into categories and themes. As a result, the themes were coded inductively using Grounded Theory by concepts that arose through content in the transcripts [13, 14]. Themes are presented descriptively using the codes with highest frequency. Finally, codes were deductively aligned back to the themes based on the concepts applied from the broader Saleema evaluation framework (e.g., Social Marketing principles). Codes were not mutually exclusive and were assigned to multiple codes if the text represented multiple concepts. A final codebook was developed by the primary coder.

The codebook was validated using a second coder (NB) on 10% of the transcripts. The second coder was also not involved with the study design or data collection. The coding comparison showed high agreement between the two coders through an average kappa of 0.80, generated using NVIVO 11.4 software. Coding discrepancies were discussed and resolved as needed by the Principal Investigator (WDE). This type of qualitative analysis technique has been utilized in various disciplines [13, 14]. Themes and exemplars italicized quotes are presented in the results. The number of coding instances, not necessarily the number of individuals, is listed next to the code only to illustrate the estimated magnitude of each theme [15].

## Results

The present qualitative evaluation included four focus groups within *N* = 18 states in Sudan. Each state included focus groups in two geographically separate localities. There were a total of 71 focus groups across all states, with roughly 5-10 individuals per group. This excluded one female group in Blue Nile for which recorded data was incomplete and consequently not included in the analysis. A tracking summary for each state is presented in Table 1. The coding results below represent high-level themes with specific codes outlined below. For each code, the results first are presented numerically in parentheses (source, reference). The source shows the magnitude of the number of sources, or number of states with the code. Then the reference is the frequency the code was endorsed across states. Quotes were not outlined by gender and locality to protect participant identities due to small sample sizes within each group.

**Table 1:** Focus Groups by State

Sudan State	Male Focus Group	Female Focus Group	Focus Group Total
Blue Nile	2	1	3
Central Darfur	2	2	4
East Darfur	2	2	4
Gadarif	2	2	4
Gezira	2	2	4
Kassala	2	2	4
Khartoum	2	2	4
North Darfur	2	2	4
North Kordofan	2	2	4
Northern State	2	2	4
Red Sea	2	2	4
River Nile	2	2	4
Sennar	2	2	4
South Darfur	2	2	4
South Kordofan	2	2	4
West Darfur	2	2	4
West Kordofan	2	2	4
White Nile	2	2	4
TOTAL	36	35	71

### Information sources and values

Participants discussed individuals and sources where they typically acquire information. The most common source of information or admiration was through leaders in the community (*n* = 16, *n* = 53), particularly religious (*n* = 17, *n* = 83) or local administrators (*n* = 18, *n* = 123). Other individuals were those in positions of authority, including family members (*n* = 14, *n* = 65) or clinicians (*n* = 13, *n* = 29). Individuals noted, “*The elderly people, the one who leads the prayers, the mayor of the neighborhood, not necessarily someone who came from outside, but anyone whose speech is accepted”* (Blue Nile) and “*Mayors and neighborhood wise men and wise women because women listen to them, and imams all people listen to them*.” (West Darfur).

The most common source of information or admiration via entertainment was through music (*n* = 11, *n* = 33). Frequent information channels used were the internet (e.g., Facebook, WhatsApp, Google), (*n* = 17, *n* = 88), telephones (*n* = 14, *n* = 95), TV (*n* = 18, *n* = 94) and radio (n = 17, n = 88), word of mouth (*n* = 13, *n* = 62), and print materials (*n* = 15, *n* = 40). One individual in West Darfur mentioned, “*Back in the days, you chat with you friends and find information, but now information comes through the internet and everyone who find new information comes and tells the others*.” However, there was some discussion of the radio or community events being more common for rural and disadvantaged areas. “*Some people their phones are not smart, so they get their information from radio and TV*” (Sennar) and “*Coffee sessions, the events in the village where the information are disseminated*” (Gadarif).

Participants were most inclined to mention they admire individuals who are responsive to their community (*n* = 12, *n* = 67) and/or have power (*n* = 10, *n* = 36). Other similar attributes included respect (*n* = 14, *n* = 43) or admiring those with talent (*n* = 9, *n* = 20). One participant in West Darfur described these trends by saying, *“A famous personality in the community especially the simple community. He is a local administration man, and he has contributions and served the community a lot. He is also a man of religion and his word is listen to, and is local man who can solve many problems with his wisdom.”* Additionally, “*He [A regional leader] has been able to achieve security, peace and stability in the region. He has been able to assert his authority in the state despite the ongoing conflict between tribes*” (East Darfur).

Common values centered on education (*n* = 17, *n* = 96), comfort or stability (*n* = 13, *n* = 42), community solidarity (*n* = 16, *n* = 91), and religious devotion (n = 13, *n* = 66). There was a theme in Central Darfur of associating family and community health as interchangeable, *“[The community is] Important because the society where people live and its stability means the stability of the families and individuals. The community is the large family.”* Similarly, another individual described, “*Education: because without education you cannot help create a health and sound community, cannot choose good friends, cannot get a good job*” (North Darfur).

### Saleema campaign brand equity and engagement

There was strong communal receptivity of and identification with the Saleema message of not circumcising women (*n* = 16, *n* = 107). One participant in the Gezira outlined this by saying, *“The village took a decision to abandon it. We are now thinking of Saleema. Now about seventy percent of the people have stopped the circumcision.”* Yet, even with many identifying with the campaign’s message of not circumcising women, there were some individuals who felt comfortable discussing their support of circumcision (*n* = 12, *n* = 33). One mother in South Darfur mentioned,

“*The idea and habit of circumcision has appeared in our society, but I will circumcise my daughter due to social stigma, because circumcision reduces sexual desire, and if the boys knew that the girl is uncircumcised they will [be] looking for her and practice adultery with her, so I will circumcised my daughter.*”

Common sources of campaign awareness came by way of media (e.g., advertising, radio) (*n* = 17, *n* = 79), word of mouth (e.g., family or community) (*n* = 13, *n* = 41), and local Saleema events (*n* = 6, *n* = 30). For instance, one individual in North Darfur stated, “*I saw a national jazz singer in a poster, wearing Saleema’s standard, I was intrigued, so I looked it out in the internet, and discovered that he and the rest of the people in the poster were Saleema’s ambassadors. Then I attended the workshop and learned more*.” Almost half of the states had individuals who mentioned they would like to be engaged with the campaign and promote the Saleema message (*n* = 7, *n* = 11). In particular, one participant from Gezira mentioned, *“As youth, any service you want it from us, we are ready to do it for you. We do not have any problem to offer you the help you need. We will stand together with you, for promoting the campaign, such as by advertising, and anything required from us, we will not hesitate to help together with the People's Committee.”*

### Saleema campaign poster content and reactions

As outlined above, common themes designed for the posters related to individuals with bright colored clothes emitting happiness through their choice to keep women Saleema and not circumcised. The branding was intended to embody change and cohesion toward anti-FGM attitudes and norms through fictitious leaders in a typical community. The themes arising out of the focus groups are presented numerically in Table 2 with exemplar quotes in Table 3. There were other themes which arose from coding but were lower in frequency and not shown.

**Table 2:** Poster Content Coding Summary

	Poster 1		Poster 2		Poster 3	
Code	States (count)	Codes (count)	States (count)	Codes (count)	States (count)	Codes (count)
Not circumcised	10	36	13	48	9	24
Happy	11	35	12	34	11	18
Merging Communities	9	17	0	0	0	0
Strong	0	0	12	39	0	0
Choice	0	0	13	39	0	0
Change	2	3	0	0	7	33

**Table 3:** Poster Coding Exemplar Quotes

Code	Exemplar Quote
Not circumcised	*Wearing Saleema’s logo, and they ae convinced to stop circumcision and that the entire community should remain whole* (Poster 1, West Darfur).
	*A mother holding her child, and she hopes her daughter to grow healthy and be Saleema* (Poster 2, South Darfur)
Happy	*People are happy the father, mother and children because they are healthy and the father is happy with his children because they are sound. I felt that my family should be as healthy, happy and beautiful as they are* (Poster 1, North Kordofan).
	*The girl is happy. Those in black are not healthy. I like her because she is Saleema and proud of that. I wish that all girls are like her* (Poster 2, North Kordofan).
	*The mother is happy because her daughter is SALEEMA* (Poster 3, Northern State).
Merging Communities	*It means to me that it matters to everyone, and people join efforts men, women and children for it* (Poster 1, South Darfur).
Strong	*The shape of women who wears the tone of Saleema establishes the message and means that the woman is strong and has a determination and can deliver her message* (Poster 2, Central Darfur).
Choice	*A happy life, of course, because she is a strong woman in her decisions, convinced by her decisions, because she has taken the right decision* (Poster 2, Gezira).
Change	*The woman in black is not Saleema but if she is convinced by the idea, she will leave her girl in hand Saleema and in this way the coming generation will be Saleema* (Poster 3, Sennar).

Not being circumcised was a prominent theme across all three posters. It was mentioned most frequently across states in poster 2 (*n* = 13, *n* = 48). Happiness was the most common theme across the posters. This theme was most discussed in poster 1 (*n* = 11, *n* = 35) and poster 2 (*n* = 12, *n* = 34). The code representing community cohesion, or “merging communities” was shown only in poster 1. However, there also seemed to be confusion (*n* = 10, *n* = 29) about what type of ceremony was occurring (e.g., circumcision ceremony or a wedding). Regarding the codes of strength and choice, poster 2 was the only one eliciting such responses. Lastly, the concept of change was most common in poster 3 (*n* = 7, *n* = 33), yet there was some confusion about the messaging across states where some thought the mother was “sad” (*n* = 8, n = 10).

More broadly, some mentioned the posters were “beautiful” and that they wanted to be like the people in them. This was shown most prominently in poster 2 (n = 8, *n* = 18). One individual in North Kordofan stated, “*I felt that my family should be as healthy, happy and beautiful as they are.*” There was also some discussions of the Saleema colors, where many had a clear interpretation that the bright colors represented being Saleema (*n* = 11, *n* = 48). One individual in North Kordofan stated, “*Those dressed in colors are uncircumcised and those in black are circumcised.*” There were only a few locations which interpreted the black as those who do not agree with Saleema (versus being circumcised). For instance, an individual in the Red Sea said, “*The ones in blacks were either didn’t hear about Saleema or they are afraid*” and conversely in East Darfur, “*The people who are wearing the campaign’s logo are convinced of the campaign’s theme.*”

### Campaign suggestions

Across states, participants most commonly suggested awareness to prevent FGM (*n* = 13, *n* = 76). We aligned this suggestion within social marketing principles (product, price, place, promotion) ad hoc to assist in organizing the suggestions from participants. Specifically, with regard to the mode in which messaging should be communicated (product), the most common theme came in the form of advocacy (*n* = 15, *n* = 72). As stated in Gezira, *“It is important to keep this project going on and you can choose four young women and four young men from this locality to be the focal points, with whom you can continue the communications, they raise our concerns and you give them ideas to convey, and to provide them with the posters to distribute.”* Additionally*,* trainings (n = 15, *n* = 55), and posters (*n* = 13, *n* = 37) were themes that arose as well. In Central Darfur one individual outlined, “*Education is the foundation and it is necessary to raise our daughters in a good way. It is important to raise them to be decent, Saleema, educated and understanding, in order to lead the society and help their people*.”

Participants said that messaging should highlight the harms of FGM or compare people who were circumcised versus not (n = 13, *n* = 35) (price). In Sennar, “*We explain to people all the consequences after circumcision like acute inflammation, accumulation of menstruation, difficulties in giving a baby and so, and we expose all this in the society.*” Participants commonly desire to hear more from doctors (*n* = 7, *n* = 22) and religious groups (*n* = 14, *n* = 37) on the topic as well as institute trainings or education in schools (*n* = 11, *n* = 33), the media (n = 14, *n* = 72), or communal settings (*n* = 15, *n* = 66) (place). In West Kordofan, “*Broadcasted programs, as most of the people in these areas depend on only on radio. They don’t have TV set due to lack of electricity*” and East Darfur,

“*After the thorough discussions and the information that we have heard for the first time, we have become totally convinced. If anything, this indicates that the community is in need of workshops, symposiums and discussion groups, which stand for the yield good results in terms of our safety and the safety for our daughters in the future*.”

With regard to whom should be the target of message, the focus groups commonly mentioned men to marry (*n* = 6, *n* = 11), midwives (*n* = 10, *n* = 28), older women (n = 10, n = 28), and people in rural villages (n = 10, *n* = 34) (promotion). In the Central Darfur, “*Women are the main cause of the spread and persistence of circumcision. The grandmothers as well insist on the circumcision of their daughters. In their view, the uncircumcised is a disgrace and shame. She will be discarded and stigmatized by words… If the women were sensitized and become active in the campaign, certainly the campaign will be successful*.” In North Kordofan, “*Changing people opinions depends to the area if in countryside the most influenced were the teachers, sheikh of the village, imam of the mosque those can raise the awareness in a simple way to explain its harms and negatives*.” Additionally, though not common in all states, youth were specifically mentioned (*n* = 3, *n* = 6) as targets as well. In Gezira, “*Your messages are very good. Just you need to have a special unit for the youth. The messages targeting them should be direct and clear*.” And East Darfur, “*We have already reached old age and what we have learnt is more than what is yet be learnt. It is the youth’s turn to act now*.”

## Discussion

The primary purpose of the current evaluation was to assess qualitative responses to the Saleema campaign. The campaign as a whole reflects an integrated strategy, employing all 4 Ps of social marketing, including place-based strategies through community-events, participatory activities in the form of public declarations to abandon FGM, mass media and digital media messages, and public advocacy. The current study was limited in scope. Results are based on qualitative focus groups assessed Sudanese perceptions of the information sources, values, and reactions to three Saleema campaign posters that were chosen to be representative of the primary campaign messages. The most common source of information or admiration was local leaders who are responsive to their community, media-based outlets, and discussions among community members. Special consideration of dissemination outlets when targeting rural areas was discussed as well provided their lack of access to technology and available resources. Participants held high value for education, community solidarity, and/or religious devotion. These findings were consistent across a majority of the states.

With respect to the primary aim of this study, we found that participants had positive opinions of Saleema and the concepts that it conveyed. Participants responded positively to the Saleema brand and branding elements in the posters and the campaign as a whole. In particular, individuals were most receptive to the bright colors and positive messaging. It seems there could be increased clarity surrounding specific activities as well as the connotations of those individuals wearing black in the posters. In addition, there was still a sizeable group across states that was vocal about their support of FGM due to social stigma. It could be useful to consider addressing specific stigma-related topics directly in future iterations of the campaign messaging.

Overall, participants had a positive response to the posters. There were some mixed responses to each individual poster, but poster 2 was most successful in eliciting interpretations aligning with the intended message design. Many understood Saleema inferred a happy life. Poster 1 was most successful in representing merging of communities. There could be more clarity about the activity in Additional file 1: poster 1 specifically to enhance effectiveness. Additional file 2: Poster 2 was most effective in eliciting interpretations of strength and choice. Additional file 3: Poster 3 was best at conveying change of FGM norms. Combined, the posters capture the full spectrum of the campaign. Though there seemed to be high brand equity, as noted previously responses indicated there were still a substantial group supporting FGM. Therefore, there is still room to expand the campaign and decrease social norms encouraging FGM.

Focus group questions also asked about suggestions for the campaign. The most common suggestion was in the form of continued awareness. The results outlined suggestions in line with Social Marketing principles (product, price, place, promotion). Advocacy, training, and posters were suggested to highlight the harms of FGM through leaders or in community settings. Individuals suggested that these activities target older women and individuals in rural villages. However, a burgeoning theme of targeting youth for support of the campaign came through as well.

The results should be interpreted in the light of its limitations. This study presents qualitative results only and may be subject to selection bias. However, the sampling strategy utilized mitigated this issue to an extent. Of course with such a sensitive topic there could also be bias with the results for those that felt comfortable to discuss FGM in front of others or more confident in their interpretation of the posters. There is one focus group missing and results are cross-sectional. Consequently, they do not represent perceptions over time. There are also potential drawbacks to the focus groups being coded with individuals not living in Sudan. Yet, the transcripts were translated by individuals who are bilingual in Sudan. Coding questions that arose about specific wording and culture were discussed and resolved prior to summarizing the results. Lastly, generalizability of the results outside of Sudan or other localities is limited. Evaluations of the Saleema campaign at other time points as well as work on FGM in other countries and populations can refine the interpretation.

## Conclusions

The results of this focus group analysis demonstrate support for future Saleema campaign efforts promoting awareness and community engagement. In particular, there was a rising theme of community involvement by engaging youth. The campaign could capitalize on partnerships with young people and those who are respected in the community (e.g., religious leaders) or continue promoting common values aligning with the support of education and community solidarity. Efforts could also engage those willing to get involved as well as continue to utilize or adapt to those channels depending on the locality. Continuing campaign efforts have promise to decrease the harms of FGM in Sudan. Moving forward, this information should also be triangulated with quantitative results to assess campaign effectiveness in relation to attitudes and social norms.

## Additional files

Additional file 1: Poster 1 - “I am not afraid of change”. (DOC 100 kb)

Additional file 2: Poster 2 - “Because I am strong in my decision”. (DOC 109 kb)

Additional file 3: Poster 3 - “Saleema”. (DOC 89 kb)

## Abbreviations

DFID: Department for International Development
FGM/C: Female Genital Mutilation and Cutting
UNICEF: United Nations International Children’s Emergency Fund
USAID: US Agency for International Development
WHO: World Health Organization
