# Supplementary figures and images for: Qualitative evaluation of the Saleema campaign to eliminate female genital mutilation and cutting in Sudan
Source: Reprod Health. 2018 Feb 17;15:30. doi: 10.1186/s12978-018-0470-2 (PMC5816556; doi:10.1186/s12978-018-0470-2)

**Additional file 1: Poster 1 - “I am not afraid of change”**


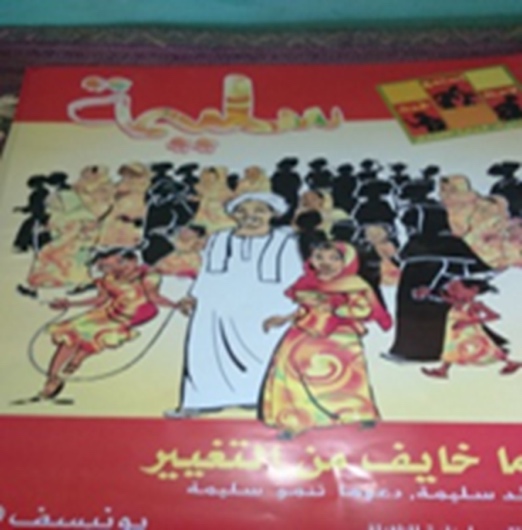

Supplement: Supplementary file 1 — Poster 1 - “I am not afraid of change”. (DOC 100 kb) [file 12978_2018_470_MOESM1_ESM.doc]

**Additional file 2: Poster 2 - “Because I am strong in my decision”**


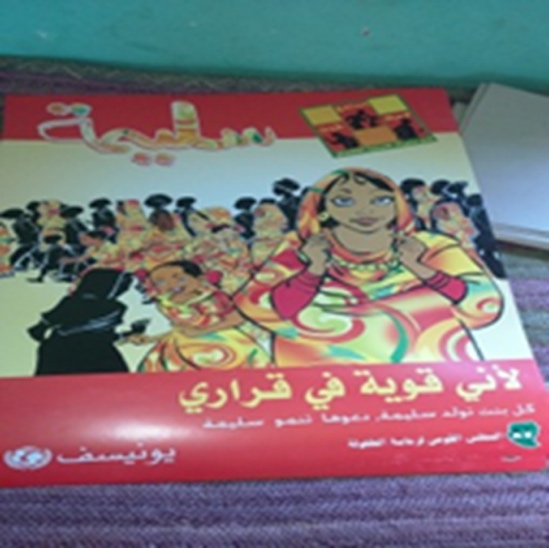

Supplement: Supplementary file 2 — Poster 2 - “Because I am strong in my decision”. (DOC 109 kb) [file 12978_2018_470_MOESM2_ESM.doc]

**Additional file 3: Poster 3 - “Saleema”**


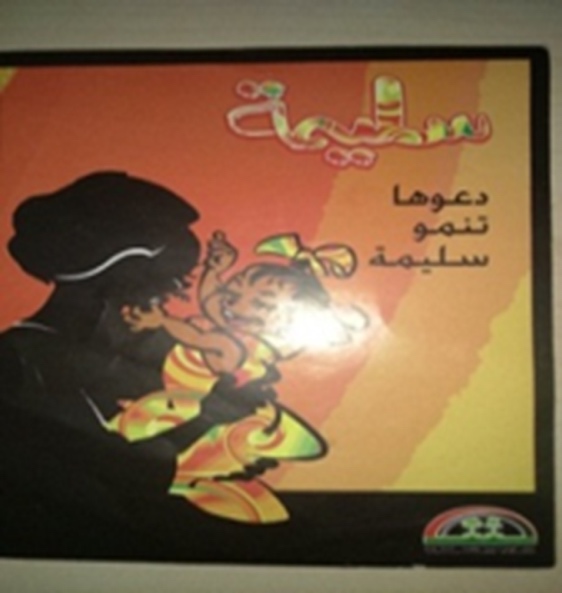

Supplement: Supplementary file 3 — Poster 3 - “Saleema”. (DOC 89 kb) [file 12978_2018_470_MOESM3_ESM.doc]
